# Perception of Risk for Developing Severe Illness or Complications from COVID-19 in Brazil: Focus on Factors Linked to Socially Vulnerable Populations, 2020–2023

**DOI:** 10.3390/ijerph22020251

**Published:** 2025-02-11

**Authors:** Rander Junior Rosa, Letícia Perticarrara Ferezin, Mônica Chiodi Toscano de Campos, Heriederson Sávio Dias Moura, Thaís Zamboni Berra, Natacha Martins Ribeiro, Titilade Kehinde Ayandeyi Teibo, André Luiz Teixeira Vinci, Antônio Carlos Vieira Ramos, Murilo César do Nascimento, Miguel Ángel Fuentealba Torres, Ricardo Alexandre Arcêncio

**Affiliations:** 1 School of Nursing, University of São Paulo at Ribeirão Preto, Ribeirão Preto 14040-902, Brazil; lehferezin@usp.br (L.P.F.); monicachiodi@unb.br (M.C.T.d.C.); heriederson@usp.br (H.S.D.M.); thaiszamboni@live.com (T.Z.B.); natacharibeiro@usp.br (N.M.R.); tayandeyi@usp.br (T.K.A.T.); altvinci@gmail.com (A.L.T.V.); ricardo@eerp.usp.br (R.A.A.); 2 Nursing Departament, Unversity of Brasília, Brasília 70910-900, Brazil; 3 Centro de Engenharias, State University of Minas Gerais, Passos 96010-440, Brazil; antonio.ramos@uemg.br; 4 College of Nursing, University of Alfenas, Alfenas 37130-001, Brazil; murilo.nascimento@unifal-mg.edu.br; 5 Faculty of Nursing and Obstetrics, Universidad de Los Andes, Santiago 12455, Chile; mafuentealba@uandes.cl

**Keywords:** associated factors, risk perception, COVID-19, pandemic

## Abstract

In this study, the aim was to comparatively examine the perception of risk for developing severe illness or complications due to COVID-19 among the general population and socially vulnerable populations in Brazil, focusing on uncovering the associated factors that disproportionately impacted people experiencing homelessness and slum dwellers. This study is part of the project “Social Thermometer—COVID-19 in Brazil”, which employed a hybrid approach, combining a national online survey with field research in state capitals and the Federal District. Data collection took place from August 2020 to October 2023, and the data were analyzed using descriptive statistics and logistic regression. A total of 5094 participants were included in this study, with 2363 from the general population and 2731 from the socially vulnerable population. Among the general population, the majority of participants were women, white individuals, those with higher incomes, formal employment, and higher education levels. Concerning the vulnerable population, most were men, Black individuals, those with lower incomes, unemployment, and lower education levels. It was observed that 87% of the general population perceived a risk of severe COVID-19, compared to 74% of the vulnerable population. Slum dwellers who received emergency aid (OR_a_ = 1.39; 95% CI: 1.02–1.91), adhered to mask-wearing practices (OR_a_ = 1.93; 95% CI: 1.39–2.66), used COVID-19-related medications (ORa = 2.13; 95% CI: 1.31–3. 64), and those with pre-existing conditions, such as high blood pressure (OR_a_ = 1.86; 95% CI: 1.20–2.98), demonstrated a heightened perception of risk for severe COVID-19 complications. Among the homeless population, individuals who wore masks (OR_a_ = 1.67; 95% CI: 1.26–2.20 and had been vaccinated (OR_a_ = 1.44; 95% CI: 1.04–1.98) were also more likely to perceive a high risk. In conclusion, in this study, significant disparities are revealed in the perception of COVID-19 risk between the general and socially vulnerable populations in Brazil. Factors such as receiving emergency aid, adherence to mask-wearing, use of COVID-19-related medications, and pre-existing health conditions were associated with increased risk perception. Despite facing greater socioeconomic challenges, vulnerable groups, particularly those experiencing homelessness and slum dwellers, showed a lower perception of the risk for severe COVID-19 complications.

## 1. Introduction

The COVID-19 pandemic profoundly impacted the global population, creating an unprecedented public health crisis [1]. Brazil, with its large population and structural inequalities, became one of the countries most affected by the pandemic. The high infection rate and an overwhelmed healthcare system, combined with the difficulty of implementing effective prevention measures in a country of continental dimensions, exacerbated the situation. The health crisis also highlighted the regional and social inequalities of the country, revealing the vulnerability of certain population groups compared to more advantaged ones [2].

The pandemic made no distinction between social classes, but it exposed even more clearly the deep disparities that mark the lives of those who already lived on the margins. The poorest communities, with less access to resources and essential services, faced an overwhelming burden that extended across various aspects of their lives [3,4]. In this sense, over the course of the pandemic, the virus spread to socially vulnerable populations, exacerbating pre-existing inequalities, particularly those related to social and structural factors.

These populations face numerous barriers that heighten their vulnerability to COVID-19, including restricted access to healthcare services, challenges in adopting preventive measures such as social distancing, and inadequate housing conditions that facilitate the spread of the virus [5,6]. Poor living conditions and a lack of basic infrastructure significantly contribute to increased exposure and amplify the pandemic’s impact on these groups [7]. These factors place them in a distinct position regarding adherence to preventive measures, risk of exposure, susceptibility to infection, and the occurrence of adverse outcomes [8].

Although COVID-19 vaccines were introduced in December 2020 as a critical public health tool, insufficient vaccination coverage among vulnerable groups may worsen disease incidence [9,10]. This situation has heightened the demand for more robust state actions in pandemic control and health care promotion, underscoring the need for an equitable approach [11].

Among socially vulnerable populations, people experiencing homelessness and residents of urban slums or disadvantaged communities were indeed more severely affected by COVID-19 and may have had their territories affected by potential syndemic effects. Possible adverse interactions between two or more health conditions or diseases occurred within these populations’ territories, exacerbated by their social and economic conditions [12].

Historically, these populations have experienced higher rates of negative health outcomes due to inequality compared to the general population, and COVID-19 has likely aggravated this situation. Syndemic effects are often amplified by social inequities, poverty, stress, and structural social determinants [13]. These contextual factors create conditions that allow diseases to cluster and interact more severely within vulnerable communities. It is important to note that while these populations are disproportionately affected by syndemics, their awareness of risk may vary. Many individuals might experience the effects without necessarily being aware of them or their implications.

For people experiencing homelessness, basic preventative measures like hand washing and social distancing became nearly impossible tasks. Due to their context of deprivation, health issues might be ignored or considered unimportant. Similarly, residents of densely populated urban slums faced heightened exposure to the virus due to overcrowded housing and limited access to sanitation. In these communities, where multiple families often share small living spaces, the concept of isolation was more aspirational than reality [14].

According to the literature, homelessness can be defined as a condition of lacking stable, safe, and functional housing, which encompasses a range of living situations. This includes people sleeping on the streets or in places not meant for human habitation, those staying in temporary accommodation or shelters, individuals or families without a regular or fixed residence, and those lacking adequate night-time residence. This comprehensive definition recognizes that homelessness extends beyond visible rough sleeping to include various forms of housing insecurity, capturing the complex and multifaceted nature of the issue [15].

Slum dwellers, differently from those experiencing homelessness, are people who live in houses in urban areas characterized by precarious housing and infrastructure conditions. These dwellings are typically built of masonry or recyclable materials. Slum areas are generally densely populated, featuring low-quality construction and limited durability of dwellings [16].

They are often marked by the absence, incompleteness, or precarious provision of essential public services, such as electric lighting, water supply, sanitation, drainage systems, and regular garbage collection. This definition highlights the urban nature of slums, the substandard living conditions, and the specific challenges faced by their residents, particularly in terms of inadequate infrastructure and basic amenities [17]. Slum dwellers, which house about 8% (20 million of people) of Brazil’s population [18], are autonomous territories established in areas lacking infrastructure where it is likely that COVID-19 helped maintain the areas’ negative impact [12].

According to recent data, there are significant gender disparities among those experiencing homelessness or living in slums in Brazil, as 88% of homeless individuals are men and only 12% are women; however, the proportion of women experiencing homelessness has been increasing mainly after the pandemic [19]. There was a notable rise in the number of women and families becoming homeless in Brazil [19]. In slum areas and poor urban communities, the gender distribution is more balanced (48.3% of men and 51.7% of women), but it is mainly women aged 15–49 who are overrepresented in urban slums and slum-like settings in Brazil [20].

The rapid spread and high mortality of COVID-19 among vulnerable populations underscore the importance of understanding how personal risk perception influences adherence to preventive behaviors, particularly among individuals with non-communicable diseases (NCDs) [21]. Sociodemographic factors shape this perception, and these populations, often uninformed or distrustful of health institutions, may fail to recognize the pandemic’s severity [22]. The lack of infrastructure in slums and close social proximity complicate social distancing and mask usage, increasing their exposure to the virus [23].

A significant gap remains in understanding the impact of social inequality on COVID-19 risk perception among vulnerable populations [24]. The lack of studies exploring how these communities construct their risk perception may lead to an underestimation of the pandemic’s true impact on these groups [25,26]. The insufficient understanding of how these individuals assess and respond to the COVID-19 threat hinders the development of effective public health interventions [27]. Therefore, in this study, the aim was to comparatively examine the perception of risk for developing severe illness or complications due to COVID-19 among the general population and socially vulnerable populations in Brazil, focusing on uncovering the associated factors that disproportionately impacted people experiencing homelessness and slum dwellers.

## 2. Materials and Methods

### 2.1. Study Design

This is a cross-sectional study with a descriptive–analytical design, part of a larger project titled “Social Thermometer—COVID-19 in Brazil”, conducted using a hybrid approach. The methodology included an online survey covering the entire national territory with the general Brazilian population and field research conducted in the capitals of the 26 federative units and the Federal District, focusing on socially vulnerable populations. Data collection took place between August 2020 and October 2023.

### 2.2. Study Population, Inclusion and Exclusion Criteria

The study population consisted of the general population of Brazil and socially vulnerable populations, specifically people experiencing homelessness and slum dwellers.

The general population consisted of individuals who self-identified as residents of Brazil, aged 18 years or older, and who had access to the questionnaire in its online version.

The socially vulnerable population in this study, represented by people experiencing homelessness and slum dwellers, included Brazilian-born or naturalized citizens aged 18 years or older who were proficient in Brazilian Portuguese. Participants included those who were homeless or had been residing in urban slum areas for at least six months during the COVID-19 pandemic. The pandemic period was defined as beginning on 11 March 2020, the date when the WHO officially declared COVID-19 a pandemic [28]. Individuals who did not respond to the study’s key variables were excluded.

### 2.3. Study Selection and Sampling

#### 2.3.1. General Population

The selection of participants for the general population was conducted using the snowball sampling technique [29,30]. Initially invited individuals were asked to complete the questionnaire and share the invitation with others within their network. This study was promoted through the websites of participating institutions, email, WhatsApp, and social media platforms (Facebook, Instagram, and Twitter), as well as through the direct involvement of researchers, who also invited people from their respective circles.

It is important to note that while the snowball sampling technique does not involve calculating margins of error or confidence levels due to its non-probabilistic nature, it is possible to estimate the sample size. For this, the calculation for finite populations was applied, considering a 5% margin of error, a 95% confidence level, 80% power, and 50% variance. Additionally, a 10% increase was added to account for potential sample losses [31]. The calculation was performed based on Brazil’s geographic division into the following macro-regions: Central-West, Northeast, North, Southeast, and South, along with their respective total populations (Table 1).

#### 2.3.2. Population in Situations of Social Vulnerability

The selection of the socially vulnerable population, characterized primarily by its invisibility to society and the state, as well as by living in a context of inequality and social exclusion, was conducted using sequential sampling [32].

The sample size calculation for the socially vulnerable population was based on data from the 2022 IBGE Census, which provided information on the number of people experiencing homelessness and slum dwellers in Brazil. In 2022, approximately 281,472 people were experiencing homelessness, while over 16.6 million people lived in slums in Brazil [18,33].

Data collection among the people experiencing homelessness took place in locations such as parks, shelters, social assistance centers, and hostels. The data collection was conducted through a one-time approach with each participant, meaning that each individual was interviewed only once, without the need for multiple sessions or additional contacts. This strategy is justified by the high mobility of these individuals, who rarely remain in the same location daily. In slum dwellers, the data collection was supported by community leaders, who acted as intermediaries. They not only facilitated access to participants but also guided the research team on the best locations and times to conduct interviews, ensuring an appropriate and safe reception.

In Table 2 below is the calculation of the minimum sample size, based on Brazil’s geographical division into the macro-regions of Midwest, Northeast, North, Southeast, and South. The table includes the total populations of these regions, and the sample sizes achieved in this study, specifically for the socially vulnerable population.

### 2.4. Instruments and Data Collection

This study used a questionnaire developed by the National School of Public Health at the Nova University of Lisbon (ENSP-UNL), which was later validated and published in studies with various objectives and perspectives by researchers from ENSP-UNL. In Brazil, the questionnaire underwent cultural adaptation and validation by senior researchers using the Delphi technique and was titled “Social Thermometer COVID-19: Social Opinion” [34].

The design of the instrument and data collection were carried out using the Research Electronic Data Capture [35,36]. (REDCap) tool at the University of São Paulo (USP), located at the Ribeirão Preto campus.

Data collection from the general Brazilian population was conducted through the dissemination of the research instrument link on the internet, via advertisements on social media platforms associated with the research and the involved researchers (such as Twitter, Facebook, WhatsApp, among others), in addition to being promoted on the institutional websites of the participating organizations.

The administration of the questionnaire to socially vulnerable populations was facilitated by the creation of a network of contacts made up of professionals from research institutions, universities, and civil society organizations. Furthermore, a mobilization strategy was implemented through Social Movements, which played a key role in establishing connections with homeless populations and slum dwellers, ensuring greater access to these communities through approaches tailored to their specific contexts.

Data were collected through face-to-face survey interviews using mobile devices (cell phones and/or tablets), who underwent rigorous training to ensure standardized instrument application and minimize measurement bias. Each interview lasted, on average, 20 to 30 min. Although some interviewers were recruited through Social Movements, this was not a requirement of this study. During the interviews, interviewers did not need an internet connection, but at the end of the data collection, they were required to access an internet point to upload the collected data to the REDCap platform.

It is worth highlighting that prior to the start of data collection, the interviewers underwent detailed training conducted by the research coordinator. The primary goal of this training was to standardize methodological approaches, providing the interviewers with clear guidelines on how to conduct interviews in a consistent manner.

This measure was essential to ensure uniformity in the data collection procedures, thereby enhancing the consistency and reliability of the data. Additionally, standardization minimized potential discrepancies in measurements, significantly reducing the risk of measurement bias during the information-gathering process.

### 2.5. Study Variables

#### 2.5.1. Dependent Variables

Table 3 presents the dependent variable of this study, used to identify the associated factors that disproportionately affected the homeless population and slum dwellers. This variable was derived from the parent study questionnaire and includes its original response patterns. Additionally, adjusted versions for binary logistic regression analyses are provided, where the data were dichotomized into values of 0 and 1.

#### 2.5.2. Independent Variables

Table 4 presents the operationalization of the independent variables of this study. It details how each variable was measured and transformed for analysis, offering a clear view of the dichotomization criteria and response categories.

### 2.6. Statistical Analysis

The data were analyzed using descriptive statistics to outline the sociodemographic and socioeconomic profile of the general population and the socially vulnerable populations in this study. The absolute (n) and relative (%) frequencies of the categorical variables were calculated. An exploratory analysis of the data was carried out to check for relationships and patterns within the data set. This phase was important to check for missing values, outliers, and inconsistencies in the data. These analyses were carried out using RStudio software, version 4.3.1.

To visually present the flow of interactions between socioeconomic and sociodemographic variables, clinical conditions, and access to healthcare services in relation to the perception of the risk of developing severe illness or complications due to COVID-19, both in the general population and in vulnerable populations, a descriptive approach was adopted using a Sankey diagram. The flows of the variable categories are represented according to the proportion of response frequencies, resulting in lines whose width is proportional to the magnitude of the data they represent. The diagram was constructed using Python software, version 3.7.7.

To carry out the binary logistic regression, the independent variables in the final regression model were selected in two stages, as follows: First, variables with *p* < 0.2 were selected in bivariate analyses through bivariate analysis using the chi-square test [37], and then the Akaike information criterion (AIC) was applied to refine the selection of the final model [38]. The crude odds ratios (ORs) were calculated along with their respective 95% confidence intervals (95% CI).

In the second stage, after including the variables in the model, multicollinearity was assessed to avoid inserting correlated variables. The presence of multicollinearity was tested using the variance inflation factor (*VIF*), one of the most widely used measures, defined by the following expression:

$${VIF}_{j}=\frac{1}{1-R_{j}^{2}}$$

where $R_{j}^{2}$ is the coefficient of multiple correlations resulting from the regression of $X_{j}$ on the other *p* − 1 regressors. The greater the degree of dependence of $X_{j}$ on the remaining regressors, the stronger the dependence and the higher the value of $R_{j}^{2}$. A cut-off value of *VIF* > 10 was adopted [39].

It is worth noting that variables with a *VIF* < 10 were included in the model. Finally, the best model was selected based on the lowest value of the AIC [40]. For the final model, the adjusted odds ratios (OR_adj_) and their respective 95% confidence intervals were calculated.

Once the best model had been estimated and chosen based on the lowest AIC value, validation tests were carried out, including the Hosmer–Lemeshow test, the likelihood ratio, the Cox–Snell test, the Nagelkerke test, and the McFadden test. For all the tests, a statistical significance level of 5% was considered. These analyses were carried out using RStudio software, version 4.3.1.

### 2.7. Ethical Aspects

This research was approved by the Research Ethics Committee of the Ribeirão Preto School of Nursing at the University of São Paulo (EERP-USP), under CAAE number: 57933622.4.1001.5393. The entire investigation was conducted in accordance with Resolution No. 466, of 12 December 2012 of the National Health Council, taking into account the relevant ethical and scientific grounds. Before starting to administer the questionnaire, the interviewers instructed the participants to read the Informed Consent Form (ICF) and sign it if they agreed to take part in the research. The ICF was drawn up in two copies, which were read in full and initialed on every page by the participant and the interviewer responsible for administering the instrument. In cases where participants were unable to read, their fingerprints were taken. The interviewees were informed that participation in the research was voluntary, meaning that they had the right to decide whether or not they wanted to take part.

## 3. Results

### 3.1. Demographics

Of the 5094 participants included in this study, 2363 belonged to the general population and 2731 to the socially vulnerable population (Table 5). In the general population, 73.3% were women, while 60.9% of individuals in vulnerable situations were men. The age group of 30 to 59 years was the majority in both groups, representing 57.4% of the general population and 65.1% of the socially vulnerable population.

Regarding race/color/ethnicity, 66.9% of the general population self-identified as White, while 74.3% of the socially vulnerable population self-identified as Black/Mixed race. In terms of marital status, 53.9% of the general population and 75.8% of the socially vulnerable population self-identified as widowed, separated, or single.

Regarding education, 88.4% of the general population had higher education, whereas 45.6% of the socially vulnerable population had completed elementary school. In terms of occupation, 51.3% of the general population reported having formal employment, while 39.4% of the socially vulnerable population reported being unemployed and 31.7% reported working informally.

Regarding income, 67.1% of the general population reported earning more than three minimum wages, while 39.0% of the socially vulnerable population lived on less than one minimum wage. Furthermore, among individuals in vulnerable situations, it was found that 55.1% were homeless, and 52.5% did not receive any type of government assistance.

**Figure 1:**
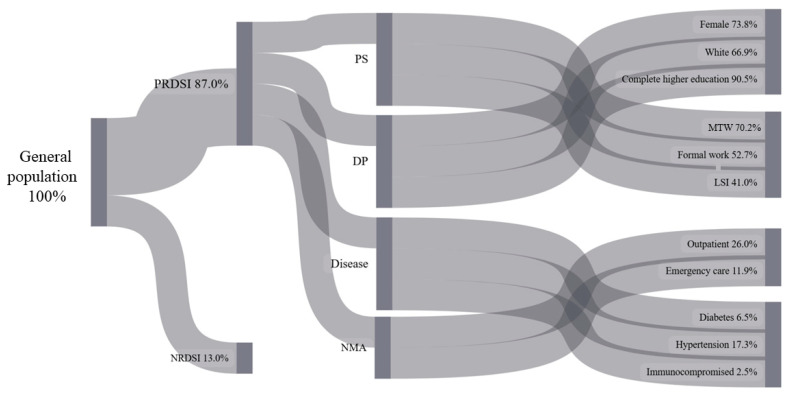
Sankey diagram showing the flow of interactions between study variables related to the perception of risk for developing severe illness or complications from COVID-19 among the general population in Brazil, 2020–2023. Legend: Perception of risk of developing serious illness or complications due to COVID-19 (PRDSI); not perception of risk of developing serious illness or complications due to COVID-19 (NRDSI); profile socioeconomic (PS); demographic profile (DP); needed medical attention (NMA); lost salary income (LSI); more than three minimum wages (MTWs). Source: analyzed and generated by authors (2025).

Figure 1 presents the interaction flows of variables related to the perception of the risk of developing severe illness or complications due to COVID-19 in the general Brazilian population, illustrated through a Sankey diagram. This diagram represents flows between different categories or variables in a clear and intuitive manner, with the width of the connections proportional to the contribution or magnitude of each flow. In other words, it integrates the variables analyzed in this study, highlighting the relationships and relative contributions of each one to the outcome under investigation.

The study population, shown in the Sankey diagram (Figure 1), was divided into the following two groups: those who perceive the risk of developing severe forms of COVID-19 (PRDSI, 87.0%) and those who do not perceive this risk (NRDSI, 13.0%).

Among those who perceive the risk, the diagram details sociodemographic characteristics (PS), healthcare service demand (DP), and health conditions (disease). In the sociodemographic profile, it is observed that 73.8% are women, 66.9% identify as White, 90.5% have completed higher education, 70.2% earn more than three minimum wages (MTWs), 52.7% have formal employment, and 41.0% did not experience income loss during the pandemic (LSI). Regarding healthcare service demand, 26.0% sought outpatient care, and 11.9% used emergency services. As for health conditions, 17.3% reported hypertension, 6.5% indicated diabetes mellitus, and 2.5% reported some form of immunocompromise. The group that did not perceive the risk of developing severe forms (NRDSI) represents 13.0% of the population.

This model highlights that women, individuals with higher education, and those without income loss represent significant portions of those who perceive the risk, while conditions such as hypertension are predominant in this group.

**Figure 2:**
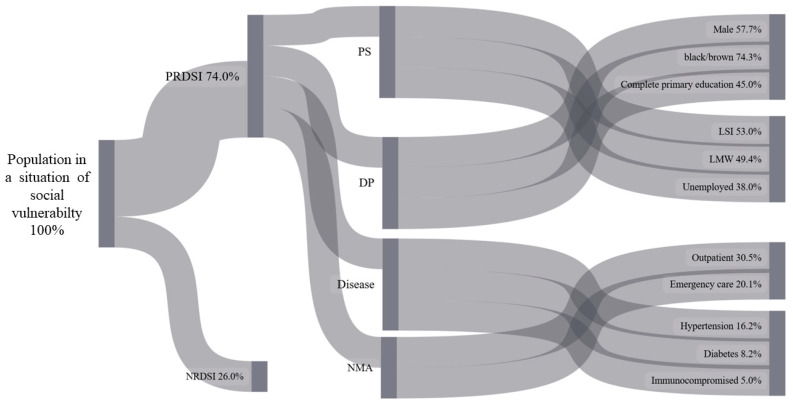
Sankey diagram showing the flow of interactions between study variables related to the perception of risk for developing severe illness or complications from COVID-19 among socially vulnerable populations in Brazil, 2020–2023. Source: analyzed and generated by authors (2025). Legend: perception of risk of developing serious illness or complications due to COVID-19 (PRDSI); not perception of risk of developing serious illness or complications due to COVID-19 (NRDSI); profile socioeconomic (PS); demographic profile (DP); needed medical attention (NMA); lost salary income (LSI); less than a minimum wage (LMW).

Figure 2 illustrates the interaction flows of the variables related to the perception of the risk of developing severe illness or complications from COVID-19 among the Brazilian population in situations of social vulnerability, represented through a Sankey diagram.

The socially vulnerable population depicted in the Sankey diagram (Figure 2) is divided into the following two groups: those who perceive the risk of developing severe forms of COVID-19 (PRDSI, 74.0%) and those who do not perceive this risk (NRDSI, 26.0%). Within the PRDSI group, variables related to social profiles are highlighted, such as 57.7% being men, 74.3% identifying as Black or Brown, 45.0% having completed only primary education, 53.0% belonging to an older age group (LSI), 49.4% falling within a lower-income bracket (LMW), and 38.0% being unemployed.

Regarding service demand, the diagram shows that 30.5% use outpatient care, while 20.1% rely on emergency services. In terms of health conditions, 16.2% have hypertension, 8.2% have diabetes, and 5.0% are immunosuppressed.

This diagram highlights the social and health inequalities faced by the socially vulnerable population, with a high prevalence of risk perception and chronic conditions such as hypertension and diabetes. It also reveals gaps in access to essential healthcare services and underscores the need for targeted monitoring and support for this population.

### 3.2. Regression Results

Table 6 and Table 7 present the results obtained from the binary logistic regressions. Table 6 shows that slum dwellers were more likely to perceive a risk of developing serious illness or complications due to COVID-19 if they reported receiving emergency aid (OR = 1.39; 95% CI: 1.02–1.91), wore a mask (OR = 1.93; 95% CI: 1.39–2.66), used medication because of COVID-19 (OR = 2.13; 95% CI: 1.31–3.64), used paracetamol (OR = 2.00; 95% CI: 1.30–3.17), used ivermectin (OR = 1.71; 95% CI: 1.07–2.86), or reported having high blood pressure (OR = 1.86; 95% CI: 1.20–2.98).

Conversely, participants who reported having a health center in their neighborhood had a lower probability of this perception (OR = 0.49; 95% CI: 0.16–0.86). The factors associated with higher risk perception are linked to a greater awareness of the severity of the pandemic and health vulnerability. However, the presence of a healthcare unit in the neighborhood is related to a lower perception of risk, possibly due to the sense of security provided by easy access to health services.

To validate the model shown in Table 6, the accuracy of the model was found to be 0.68 using the area under the ROC curve, the Hosmer–Lemeshow test (*p* = 0.24), the likelihood ratio (*p* < 0.01), Cox–Snell (0.07), Nagelkerke (0.11), and McFadden (0.06).

Table 7 showed that among people experiencing homelessness, those who wore a mask (OR = 1.67; 95% CI: 1.26–2.20), had been vaccinated against COVID-19 (OR = 1.44; 95% CI: 1.04–1.98), had diabetes mellitus (OR = 2.84; 95% CI: 1.28–7.58), hypertension (OR = 2.11; 95% CI: 1.18–4.07), or immunocompromised disease (OR = 4.44; 95% CI: 1.92–12.9) were more likely to perceive a risk of developing serious illness from COVID-19. These findings highlight how health conditions and adopted preventative measures influence risk perception among people experiencing homelessness. The use of masks and vaccination, while protective measures, are also associated with a greater awareness of risk. Furthermore, the presence of chronic or immunosuppressive diseases elevates the perceived risk of developing serious illness or complications due to COVID-19.

To validate the model shown in Table 7, the accuracy of the model was found to be 0.65 using the area under the ROC curve, the Hosmer–Lemeshow test (*p* = 0.13), the likelihood ratio (*p* < 0.01), Cox–Snell (0.05), Nagelkerke (0.08), and McFadden (0.05).

## 4. Discussion

In this study, the aim was to comparatively examine the perception of risk of developing severe illness or complications due to COVID-19 among the general population and socially vulnerable populations in Brazil, with a focus on uncovering the associated factors that disproportionately impacted people experiencing homelessness and slum dwellers. It was observed that 87% of the general population perceived a risk of severe COVID-19 compared to 74% among the vulnerable population. Regarding the profile of the general population, the majority were women, white individuals, with higher incomes, formal employment, and higher education levels. Concerning the vulnerable population, most were men, Black individuals, with lower incomes, unemployment, and lower education levels.

### 4.1. Differences in Risk Perception Between the General Population and Socially Vulnerable Populations

Differences regarding risk perception have been shown in the literature, aligning with our findings. A study conducted in Germany also showed that people with higher levels of education demonstrated greater factual knowledge about COVID-19 and expressed more concern regarding the disease [41]. Furthermore, men with higher levels of education demonstrated greater concern about COVID-19 compared to those with lower levels of education [41]. One difference is that the study revealed that the majority were men, unlike our investigation which primarily involved women.

Conversely, socially vulnerable populations typically experience lower risk perception, influenced by barriers to accessing information and healthcare services [42]. Our findings corroborate this, revealing that limited education and income levels can hinder access to reliable health information, delay disease recognition, and reduce adherence to preventive measures [43]. During the pandemic’s critical phase, these groups faced compounded challenges, such as dependence on public transportation and restricted healthcare access, which likely exacerbated their lower risk perception.

A study also revealed that among university students, particularly those in the medical field, more rational and informed perceptions about COVID-19 risks were observed due to their academic training. They were more likely to adopt preventative behaviors [44]. In contrast, a study showed that people in situations of greater socioeconomic vulnerability tend to have a lower risk perception regarding COVID-19 [42]. They were more vulnerable groups in having a higher chances of infection, but lower risk perception.

Our findings revealed a very low evaluation of risk, as well as delays in recognizing and treating the disease, rapid spread of the infection, and lower adherence to preventative measures. It can therefore be inferred that education level, income, and social status play a significant role in the perception of risk regarding COVID-19, influencing both the understanding of its severity and the adoption of preventive behaviors associated with the disease. Low education levels result in limited access to quality healthcare services and reliable information about COVID-19, thereby increasing the risks of illness and death [42,43].

During the pandemic, particularly in its critical phase when vaccines were not yet available, vulnerable populations faced significant challenges in practicing social isolation. Many needed to maintain employment/income by relying on public transportation, in addition to having limited access to healthcare, which might explain a lower perception of risk when compared to general population [43].

### 4.2. Behavioral and Contextual Influences on Risk Perception

Among slum dwellers, risk perception was elevated in individuals receiving emergency aid, using masks, or with pre-existing conditions such as hypertension. Evidence suggests that engagement with health services or participation in emergency programs enhances awareness of health risks [45]. Similarly, mask usage has been linked to increased risk perception, as such preventive behaviors often signal heightened awareness of health threats [46].

For homeless populations, vaccination emerged as a key factor influencing risk perception. While vaccines provide robust protection against severe COVID-19, awareness of residual risks, including transmissible variants and incomplete vaccination schedules, appears to amplify perceived vulnerability. These findings align with studies highlighting the prevalence of pulmonary diseases, such as tuberculosis, among homeless individuals, which further contributes to their heightened perception of health risks [47]. Notably, among slum dwellers who received emergency aid, adhered to mask-wearing practices, used COVID-19-related medications (including paracetamol and ivermectin), and those with pre-existing conditions like high blood pressure demonstrated a heightened perception of risk for severe COVID-19 complications.

Among the homeless population, individuals who wore masks were also more likely to perceive a high risk. The presence of comorbidities was strongly associated with an increased perception of risk. Unlike slum dwellers, homeless individuals who were vaccinated showed greater awareness regarding risk. These findings, supported by significant odds ratios, suggest that certain behaviors and health conditions may influence risk awareness, regardless of social vulnerability [48].

These results emphasize the need for integrated public health strategies that go beyond vaccination to address broader social determinants of health. Efforts should focus on improving access to healthcare, managing comorbidities, and reducing systemic inequities to mitigate vulnerabilities among socially disadvantaged groups.

### 4.3. Comorbidities and Their Impact on Perceived Risk

In this study, it was revealed that risk perception is also linked to pre-existing health conditions among the socially vulnerable population, such as hypertension, diabetes, and immunocompromised conditions. The observation that comorbidities heighten participants’ risk perception aligns with prior research showing that individuals with pre-existing health conditions are more attuned to the dangers of COVID-19 due to their increased likelihood of severe complications if infected [49]. Chronic illnesses are consistently identified in the literature as significant predictors of vulnerability, underscoring their role as critical determinants in assessing susceptibility to the virus [50].

A study demonstrated that 72% of individuals with diabetes and COVID-19 required admission to the Intensive Care Unit, compared to patients without diabetes. The findings indicated that the infection has a significant impact on individuals with comorbidities, triggering systemic inflammation. As such, diabetes mellitus was identified as a critical risk factor during the pandemic, contributing to the worsening of symptoms and leading to unfavorable health outcomes [51].

Chronic conditions such as hypertension, diabetes, and immunocompromised states were closely associated with increased risk perception among vulnerable groups. These comorbidities are well-documented contributors to heightened vulnerability, shaping individuals’ susceptibility to COVID-19 complications [43]. For instance, diabetes has been linked to a higher likelihood of severe illness, as evidenced by elevated ICU admission rates among diabetic COVID-19 patients [51].

These findings underscore the importance of incorporating chronic disease management into comprehensive risk communication strategies. By addressing the unique vulnerabilities posed by these conditions, public health interventions can enhance risk awareness and support preventive behaviors, particularly in socially disadvantaged populations.

### 4.4. Medication Use and Misinformation

Another curious finding was the use of medications such as paracetamol and ivermectin, which was positively correlated with risk perception, suggesting that these individuals were more attentive to the signs of the disease, increasing their likelihood of recognizing their own risk. In the absence of widely recognized treatments, the antiparasitic drug ivermectin gained popularity for its potential use against COVID-19. However, robust scientific evidence does not support its clinical effectiveness in treating the disease. Recent studies, including large-scale randomized trials, have consistently found no significant benefits of ivermectin in reducing symptom duration, hospitalization rates, or mortality among COVID-19 patients [52]. Unfortunately, our study highlighted instances where individuals were subjected to treatments lacking robust scientific evidence, potentially increasing their risk of developing health complications unrelated to COVID-19. This underscores the importance of relying on evidence-based medical practices to avoid unintended harm and ensure patient safety.

Additionally, COVID-19 vaccination was also identified as a protective measure associated with higher risk perception among people experiencing homelessness, a finding that may seem paradoxical at first. The vaccine provides significant protection against severe forms of the disease but does not completely eliminate the risk of infection, mainly due to the emergence of more transmissible variants and in individuals with comorbidities. Studies have suggested that greater awareness of the benefits of vaccination can lead to a deeper understanding of the risks still present, even after immunization [53,54,55].

Adding to the discussion is the fact of incomplete vaccination schedules, with many people not having received all the necessary doses to ensure their effective protection. This may be occurring among people experiencing homelessness, therefore heightening their perception of risk. Other issue is the higher risk perception among vaccinated individuals may reflect their awareness of the social and health conditions they face, such as limited access to medical care, comorbidities, and poor living conditions, which are common among people experiencing homelessness. The literature provides evidence that vulnerable individuals, such as those experiencing homelessness, are at greater risk of severe pulmonary infectious diseases, including tuberculosis, which is particularly prevalent in this population. This heightened awareness may contribute to their perception of an increased risk of developing severe illness or complications related to COVID-19 [47,56,57]. The literature is really limited in terms of interactions between tuberculosis and COVID-19, mainly in the context of vulnerability and inequality. Therefore, it is essential that public health strategies address both vaccination and the social conditions that exacerbate risks for these populations.

### 4.5. Implications for Public Health Policies

Reducing disparities in risk perception requires targeted public health interventions that prioritize education, improve healthcare accessibility, and strengthen risk communication strategies. Enhancing health literacy is essential for enabling vulnerable populations to adopt preventative measures and access healthcare services effectively. Initiatives should focus on vaccination, chronic disease management, and combating misinformation to support informed perceptions and behaviors [58,59].

For slum dwellers and homeless populations, tailored approaches must address specific challenges such as restricted healthcare access, comorbidities, and poor living conditions. Public health programs should integrate vaccination campaigns with broader efforts to address social determinants of health, reduce inequities, and mitigate systemic barriers. These strategies are fundamental not only for minimizing the immediate impacts of COVID-19 but also for enhancing resilience to future public health emergencies [60].

### 4.6. Limitations

One of the main limitations of this study was the use of non-probabilistic sampling, which may have introduced biases in participant selection, affecting the representativeness of the sample. This means that the results may not accurately reflect the diversity and realities of the populations in question, whether general or vulnerable. The lack of randomness in the sampling limits the generalizability of the findings and makes comparison with other studies more difficult.

Another limitation of this study is the potential for recall bias among participants, as interviews were conducted between August 2020 and October 2023. To minimize this effect, we employed a validated and culturally adapted instrument for the Brazilian context, featuring structured questions designed to prompt recall of key moments during the pandemic. Nonetheless, we acknowledge that responses may be influenced by selective memory or subjective interpretation, which are inherent challenges in retrospective studies. Despite these limitations, the data offer a reliable perspective on disparities in the perceived risk of severe COVID-19 complications, particularly among the general population and socially vulnerable groups.

The results of this study have significant implications for public health policies in contexts of social vulnerability, such as slums and people experiencing homelessness. Risk perception is shaped by factors including access to medical care, comorbidities, and the availability of preventive measures. To improve responses in vulnerable areas, it is essential to enhance education about risks and expand access to appropriate healthcare resources. The gap in risk perception between these populations and those with better healthcare access underscores the need for more targeted strategies. Furthermore, interventions addressing comorbidities and promoting protective measures—such as mask use and vaccination—can increase awareness, reduce the risk of severe complications, and improve health outcomes. These approaches offer a more effective framework for managing future public health crises.

## 5. Conclusions

In this study, it was evidenced that there is a notable difference in COVID-19 risk perception between the general population and socially vulnerable groups in Brazil. The general population showed a higher risk perception (87%) compared to vulnerable populations (74%), despite the latter facing greater socioeconomic challenges. In this study, distinct demographic profiles between the general and vulnerable populations were revealed, with the general population predominantly comprising women, White individuals, and those with higher socioeconomic status, while the vulnerable population consisted mostly of men, Black individuals, and those with lower socioeconomic status. For slum dwellers, receiving emergency aid, adhering to mask-wearing, using COVID-19 medications, and having pre-existing conditions like high blood pressure were associated with increased risk perception. Among the homeless population, mask-wearing and vaccination were linked to higher risk perception. The association between emergency aid receipt and increased risk perception suggests that SUS has contributed to raising awareness about COVID-19 risks, mainly among vulnerable populations. The association between mask-wearing, vaccination, and higher risk perception highlights the potential dual role of these measures in both protection and risk awareness. This is due to efforts from the SUS in reaching the population in various territories through the Family Health Strategy/Primary Health Care and Street Clinics. These conclusions highlight the complex interplay between socioeconomic factors, health behaviors, and risk perception in the context of the COVID-19 pandemic. They underscore the necessity for nuanced, targeted approaches in public health strategies to effectively address the needs and perceptions of diverse population groups, particularly those who are more marginalized and have their rights as citizens refused or denied.

## Figures and Tables

**Table 1:** Minimum sample to be enrolled from each Brazilian state (2020–2023).

Region	General Population	Minimum Sample by Macro-Region	Sample by Macro-Region Reached in This Study
West center	≅47,657,274	385	140
Northeast	≅57,071,654	385	442
North	≅18,430,980	385	260
Southeast	≅88,371,433	385	1249
South	≅29,975,984	385	272

Source: https://documents1.worldbank.org/curated/en/691661573116715073/pdf/FY2019-Brazil-Country-Opinion-Survey-Report.pdf (accessed on 26 August 2024).

**Table 2:** Minimum sample to be recruited from each Brazilian macro-region (2020–2023).

Region	People Experiencing Homelessness and Slum Dwellers	Minimum Sample by Macro-Region	Sample by Macro-Region Reached in This Study
West center	≅510,000	384	397
Northeast	≅3,560,000	585	814
North	≅1,515,000	385	519
Southeast	≅6,600,000	385	643
South	≅1,030,000	385	358

Source: https://www.ipea.gov.br/portal/categorias/45-todas-as-noticias/noticias/13457-populacao-em-situacao-de-rua-supera-281-4-mil-pessoas-no-brasil (accessed on 26 August 2024).

**Table 3:** Operationalization of the dependent variable under study.

Dependent Variables	Original Response Options from Questionnaire	Dichotomization
Risk perception of developing severe illness or complications due to COVID-19	I. High risk II. Moderate risk III. Low risk IV. No risk V. Prefer not to answer	0 = No risk
		1 = High risk, moderate risk or low risk

**Table 4:** Operationalization of this study’s independent variables.

Independent Variables	Original Answer Options for the Question or Answer Category	Dichotomization
**Age**	18 to 29 years old	0 = No
		1 = Yes
	30 to 59 years old	0 = No
		1 = Yes
	60 years or older	0 = No
		1 = Yes
**Sex/Gender**	Male Female	0 = Female 1 = Male
**Race/Color/Ethnicity**	White	0 = No
		1 = Yes
	Black/brown	0 = No
		1 = Yes
**Marital status**	Married or in a stable union	0 = No
		1 = Yes
	Widowed, separated or single	0 = No
		1 = Yes
**Employment/Occupation**	Student	0 = No 1 = Yes
	Public employee Private sector employee Microentrepreneur Domestic worker Business owner	0 = No 1 = Formal work
	Self-employed Business owner Family farmer Informal work/casual work	0 = No 1 = Informal work
	Unemployed	0 = No 1 = Yes
	Pensioner	0 = No 1 = Yes
**Education**	No schooling	0 = No 1 = Yes
	Incomplete elementary education to complete elementary education	0 = No 1 = Yes
	Incomplete secondary education to complete secondary education	0 = No 1 = Yes
	Higher education complete or more	0 = No 1 = Yes
**Use of the Unified Health System (SUS—*Sistema Único de Saúde*)**	Uses the SUS Does not use SUS	0 = No 1 = Yes
**Visit from Community Health Agent (ACS—*Agente Comunitário de Saúde*)**	Receives visit from ACS Does not receive visit from ACS	0 = No 1 = Yes
**Use of a mask**	Yes, but only in certain situations Yes, whenever I am away from home/temporary shelter I don’t use it because I find it uncomfortable I don’t use it because I think it doesn’t protect	0 = I don’t use it because I find it uncomfortable and I don’t use it because I think it doesn’t protect 1 = Yes, but only in certain situations and I don’t use it because I think it doesn’t protect
**Started or increased use of medication because of COVID-19**	Azithromycin	0 = No 1 = Yes
	Dipyrone	0 = No 1 = Yes
	Ibuprofen	0 = No 1 = Yes
	Hydroxychloroquine	0 = No 1 = Yes
**Presence of CNCDs**	Chronic kidney disease	0 = No 1 = Yes
	Chronic lung disease	0 = No 1 = Yes
	Diabetes mellitus	0 = No 1 = Yes
	Obesity	0 = No 1 = Yes
	Immunocompromised disease	0 = No 1 = Yes
	Hypertension	0 = No 1 = Yes
**Required outpatient care**	-	0 = No 1 = Yes
**Needed care in the emergency/urgent care unit**	-	0 = No 1 = Yes

**Table 5:** Characteristics of the general population and populations in situations of social vulnerability included in this study, Brazil, 2020–2023 (n = 5094).

Variables	General Population (n = 2363)		Population in Situations of Social Vulnerability (n = 2731)	
	n	%	n	%
**Sex**				
Male	621	26.3	1664	60.9
Female	1728	73.1	1012	37.1
Others	7	0.3	54	2.0
No reply	7	0.3	1	0.0
**Age (years)**				
18 to 29	565	23.9	711	26.0
30 to 59	1355	57.4	1777	65.1
60 years or older	443	18.7	243	8.9
**Race/** **Color/Ethnicity**				
White	1578	66.8	616	22.6
Black/Mixed race	749	31.7	2029	74.3
No reply	36	1.5	86	3.1
**Marital status**				
Married or in a stable union	1088	46.1	659	24.2
Widowed, separated or single	1270	53.7	2071	75.8
No reply	5	0.2	1	0.0
**Education**				
No study	4	0.2	99	3.5
Complete primary education	92	3.8	1244	45.6
Completed high school	177	7.5	1048	38.4
Complete university degree or post-graduate degree	2081	88.1	338	12.4
No reply	9	0.4	2	0.1
**Employment/Occupation**				
Formal work	1212	51.3	381	14.0
Informal work	247	10.5	867	31.7
Unemployed	111	4.7	1076	39.4
Student	403	17.1	127	4.7
Pensioner	287	12.1	150	5.5
Other	101	4.2	129	4.7
No reply	2	0.1	1	0.0
**Monthly** **i** **ncome**				
Without Income	30	1.3	657	24.1
Less than 1 minimum wage	107	4.5	1066	39.0
From 1 to 2 minimum wages	252	10.7	564	20.7
From 2 to 3 minimum wages	231	9.8	142	5.2
Above 3 minimum wages	1578	66.7	83	3.0
No reply	165	7.0	219	8.0
**Territory of vulnerability in which you live**				
Homeless	-	-	1504	55.1
Slum dwellers	-	-	1227	44.9
**Receives government aid**				
Yes	256	10.8	1293	47.3
No	1497	63.4	1435	52.5
No reply	610	25.8	3	0.2

**Table 6:** Association between sociodemographic and clinical characteristics and the perceived risk of developing severe illness or complications due to COVID-19 among slum dwellers in Brazil, 2020–2023 (n = 1227).

Variables		OR_crude_	95% CI	*p*-Value	OR_adj_	95% CI	*p*-Value
18 at 29 years	No Yes	Ref * 0.72	- (0.54–0.97)	- 0.027 ‡	-	-	-
Male	No Yes	Ref 0.71	(0.53–0.94)	0.013 ‡	-	-	-
Female	No Yes	Ref 1.35	(1.01–1.78)	0.029 ‡	-	-	-
Receives government aid	No Yes	Ref 1.42	- (1.04–1.94)	- 0.021 ‡	Ref 1.39	- (1.02–1.91)	- 0.039 *
Health center in the neighborhood	No Yes	Ref 0.52	- (0.27–0.92)	- 0.022 ‡	Ref 0.49	- (0.26–0.86)	- 0.018 *
Wearing a mask	No Yes	Ref 2.14	- (1.54–2.95)	- <0.000 ‡	Ref 1.93	- (1.39–2.66)	- <0.000 ***
Increased or started taking medication because of COVID-19	No Yes	Ref 2.89	- (1.75–5.01)	- <0.000 ‡	Ref 2.13	- (1.31–3.64)	- < 0.003 **
Use of Ibuprofen	No Yes	Ref 2.33	- (1.42–4.00)	- <0.000 ‡	-	-	-
Use of paracetamol	No Yes	Ref 2.23	- (1.44–3.56)	- 0.000 ‡	Ref 2.00	- (1.30–3.17)	- <0.002 ***
Use of hydroxychloroquine	No Yes	Ref 2.18	- (1.17–4.44)	- 0.000 ‡	-	-	-
Use of Azithromycin	No Yes	Ref 1.78	- (1.10–2.99)	- 0.010 ‡	-	-	-
Use of Ivermectin	No Yes	Ref 2.19	- (1.36–3.67)	- 0.014 ‡	Ref 1.71	- (1.07–2.86)	- 0.029 *
Use of multivitamins	No Yes	Ref 1.78	- (1.11–2.96)	- 0.013 ‡	-	-	-
Medical care by specialists (cardiologist and gynecologist)	No Yes	Ref 1.68	- (1.12–2.56)	- 0.000 ‡	Ref 1.40	- (0.93–2.13)	- 0.107 *
Diabetes Mellitus	No Yes	Ref 2.79	- (1.43–6.08)	- 0.000 ‡	Ref 1.92	- (1.00–4.09)	- 0.063 *
Arterial hypertension	No Yes	Ref 2.25	- (1.45–3.58)	- 0.047 ‡	Ref 1.79	- (1.16–2.85)	- 0.010 *
Immunocompromised	No Yes	Ref 3.12	- (0.97–15.9)	- 0.040 ‡	Ref 3.02	- (1.03–12.8)	- 0.070 *

Legend: OR_crude_: crude odds ratio; 95% CI: 95% confidence interval; OR_adj_: adjusted odds ratio. Reference level. * Significant level at *p* < 0.05, ** significant level at *p* < 0.01, *** significant level at *p* < 0.001. ‡ Variables with *p* < 0.2 (bivariate analyses) were introduced into the model. The final model was obtained using AIC: * significant level at *p* < 0.05, ** significant level at *p* < 0.01, *** significant level at *p* < 0.001.

**Table 7:** Association between sociodemographic and clinical characteristics and the perceived risk of developing severe illness or complications due to COVID-19 among the population experiencing homelessness in Brazil, 2020–2023 (n = 1504).

Variables		OR_crude_	95% CI	*p*-Value	OR_adj_	95% CI	*p*-Value
Male	No Yes	Ref * 0.67	- (0.50–0.89)	- 0.005 ‡	Ref 0.77	- (0.55–1.06)	- 0.115
Female	No Yes	Ref 1.41	- (1.04–1.92)	- 0.021 ‡	-	-	-
Singles	No Yes	Ref 0.65	(0.42–0.99)	0.042 ‡	-	-	-
Married	No Yes	Ref 1.51	- (1.00–2.35)	- 0.042 ‡	Ref 1.22	- (0.77–1.98)	- 0.391
Use of mask	No Yes	Ref 1.49	- (1.16–1.91)	- 0.001 ‡	Ref 1.67	- (1.26–2.20)	- <0.000 ***
COVID-19 vaccine	No Yes	Ref 1.64	- (1.20–2.22)	- 0.000 ‡	Ref 1.44	(1.04–1.98)	0.025 *
Increased or started taking medication because of COVID-19	No Yes	Ref 1.54	- (0.98–2.49)	- 0.048 ‡	Ref 1.23	- (0.79–1.97)	- 0.366
Use of Ibuprofen	No Yes	Ref 1.70	- (0.98–3.08)	- 0.046 ‡	-	-	-
Use of Dipyrone	No Yes	Ref 1.53	- (1.04–2.29)	- 0.025 ‡	-	-	-
Uso de Azitromicina	No Yes	Ref 2.00	- (1.01–4.31)	- 0.036 ‡	-	-	-
Medical care by specialists (cardiologist and gynecologist)	No Yes	Ref 2.06	- (1.39–3.11)	- 0.000 ‡	Ref 1.50	- (0.95–2.45)	- 0.089
Diabetes Mellitus	No Yes	Ref 2.91	- (1.29–7.70)	- 0.006 ‡	Ref 2.84	- (1.28–7.58)	- 0.018 *
Arterial hypertension	No Yes	Ref 2.56	- (1.45–4.84)	- 0.000 *‡	Ref 2.11	- (1.18–4.07)	- 0.017 **
Immunocompromised	No Yes	Ref 4.08	- (1.74–11.6)	- 0.000 ‡	Ref 4.44	- (1.92–12.9)	- <0.001 ***

Legend: OR_crude_: crude odds ratio; 95% CI: 95% confidence interval; OR_adj_: adjusted odds ratio. Reference level. * significant level at *p* < 0.05, ** significant level at *p* < 0.01 and *** significant level at *p* < 0.001. ‡ Variables with *p* < 0.2 (bivariate analyses) were introduced into the model. The final model was obtained using AIC: * significant level at *p* < 0.05, ** significant level at *p* < 0.01, *** significant level at *p* < 0.001.

## Data Availability

All the data supporting the study findings are within the manuscript. Additional detailed information and raw data will be shared upon request addressed to the corresponding author [34].
